# Social Media is Addictive and Influences Behavior: Should it Be Regulated as a Digital Therapeutic?

**DOI:** 10.2196/43174

**Published:** 2023-01-26

**Authors:** Eric Perakslis, Yuri Quintana

**Affiliations:** 1 Duke University School of Medicine Durham, NC United States; 2 Duke Clinical Research Institute Durham, NC United States; 3 Beth Israel Deaconess Medical Center Boston, MA United States; 4 Harvard Medical School Harvard University Boston, MA United States; 5 Homewood Research Institute Guelph, ON Canada

**Keywords:** social media, mental health, suicide, health policy, addictions, youth mental health, FDA, Food and Drug Administration, Canada, United Kingdom, United States, European Union, privacy, security, adverse event

## Abstract

Recently, we were deeply saddened by the findings of the coroner investigating the death of 14-year-old Molly Russell. Deeply saddened and angry but not surprised. This case should be seen as a sentinel event, given that this is the first time social media was directly implicated as a cause of death. We should use this opportunity to advance proposals for the regulations of the health effects of social media.

## Introduction: What Happened to Molly

Molly Russell died of suicide in November 2017 [1]. Just as the gun lobby and our struggling political system have failed to enact adequate protections against mass shootings in the United States, especially at schools, social media companies continue to spawn extensive mental health and physical harm, offering little more than #thoughtsandprayers in response [2,3]. Since then, the British authorities have studied her case deeply to understand the contributions of her social media use to her depression, suicidality, and death. The child psychiatrist assigned to the case, Dr Navin Venugopal, reported being unable to sleep due to the nature of the material Molly had viewed online [4]:

There were periods where I was not able to sleep well for a few weeks, so bearing in mind that the child saw this over a period of months, I can only say that she was [affected], especially bearing in mind that she was a depressed 14-year-old. It would certainly affect her and made her feel more hopeless.

**Figure 1:**
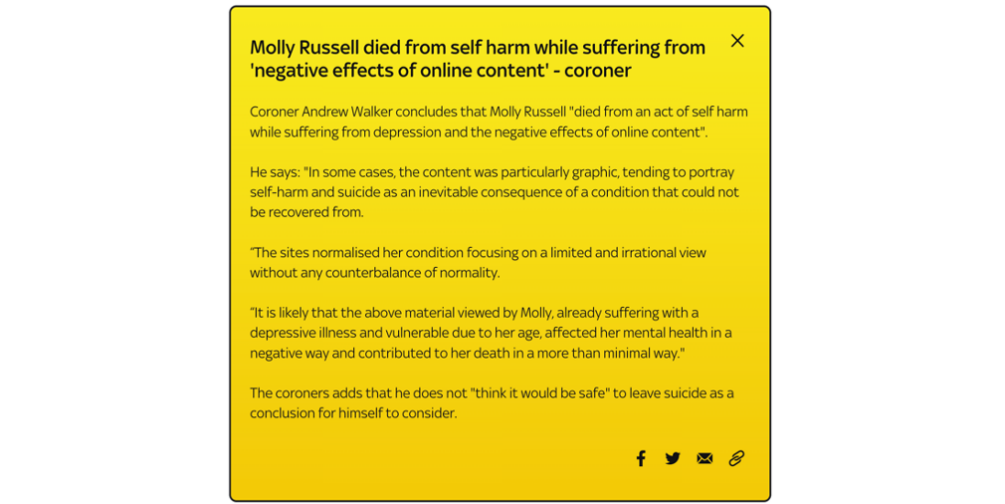
Summary of Molly Russell Coroner statements [5].

A few specific statements from coroner Andrew Walker are shown in Figure 1 via Sky News; additionally, the coroner declared the following [5]:

Molly subscribed to a number of online sites. She had access to images, video clips and text concerned with self-harm and suicide or that were otherwise negative or depressing in nature.

He went on to describe that the algorithms used by social media sites Instagram and Pinterest meant some content was selected and provided for Molly without her requesting it. The content she then kept watching more and more was likely to have harmed the teenager and “contributed to her death in a more than minimal way” [5]. These striking statements must be the catalyst for protecting children, adolescents, and adults from the harms of social media. This is not about one tragic case; it is about the clear impacts of the industry on our children.

## Current Understandings of the Harms of Social Media

Medical societies and the public have been slow to understand the depth and breadth of the harmful impact of highly targeted content on the internet, as it is often easier to visualize potential benefits than quantify the corresponding harms [6,7]. Not only does the understanding of harms significantly lag the introduction of novel technologies, but public safety regulations lag even longer, as most regulatory regimes are only authorized to regulate products within very explicit and specific product codes. In the case of social media, extensive work has been done that demonstrates links between social media use and depression, suicide attempts, and health-threatening addiction in adolescents [8-10]. Yet social media remains essentially unregulated from the standpoint of protecting consumers from harm in most of the world. This has resulted in dozens of civil lawsuits from families claiming harm that ranges from the causality of depression and anorexia to the causality of child pornography when youngsters are encouraged to share nude photos of themselves during online interactions [11].

The evidence continues to grow, and the specific mechanisms of harm are becoming more apparent. Studies of repetitive negative thinking and social media addiction positively correlate with suicide-related outcomes [12]. Other mechanisms of social media pathologies are related to addiction, impulsiveness, inhibitory control, executive function, motivations, and others [13,14].

## Digital Therapeutics Purpose, Mechanisms, and Regulation

In the European Union, digital therapeutics (DTx) are defined as evidence-based therapeutic interventions driven by software to prevent, manage, or treat a medical disorder or disease. They are patient-facing software applications that help patients treat, prevent, or manage a condition and have a proven clinical benefit [15]. The United States has no equivalently clear definition on the Food and Drug Administration (FDA) website [16]. DTx are commonly studied as a form of mobile medical apps and are regulated as software functionality that meets the definition of a medical device [17]. This makes it challenging to distinguish DTx from other health and wellness products in the United States. A pioneer in DTx, Pear Therapeutics has approved or cleared products for substance abuse disorder, opioid use disorder, and chronic insomnia [18]. The FDA has also authorized the prescription of DTx for pediatric attention deficit/hyperactivity disorder [19], major depressive disorder, and generalized anxiety disorder [20]. However, thousands of unregulated apps have appeared for mental health and wellness and make claims not backed by scientific studies to evaluate efficacy [21]. Websites are also emerging with words such as “accredited reviews,” but no accredited medical societies are involved in those reviews [22,23]. Data security in mental health apps is also a significant concern [24]. Some apps have shared the user’s data with third parties without consent [25]. It is like the early Wild West regarding apps and social media, with few regulations to protect the public, especially children.

Some apps deliver cognitive behavioral therapy (CBT) [26], a structured and goal-oriented form of talk therapy, in conjunction with psychological and psychiatric care. Internet-based CBT (iCBT) has been studied extensively, demonstrating that licensed health care professional–guided iCBT is more effective than unguided iCBT in severe and moderate depression [27-30]. There have been very few randomized control trials of iCBT applications, and most providers and the public do not have a reliable source to identify which apps have been scientifically evaluated.

Examining the mechanisms of action of DTx, some may use gamification, such as offering rewards for completion of in-app tasks or attention bias modification based on the premise that a core driver of stress and anxiety is an unconscious attentional bias toward negative or threatening information [31]. Although gamification can be used to develop “serious games for health” for improving health behaviors [32], these approaches also need to be validated to ensure they achieve therapeutic goals and not mislead users in how their data are used. A personality test app that obtained thousands of personal data from Facebook users led to the Cambridge Analytica scandal [33]. Despite a financial fine being leveled by the European Union, there is no assurance this will not occur again.

## Social Media Use as Self-Care

There have been many studies on the harms of social media, but there is also a growing body of evidence that social media can be beneficial. Social media can be anonymous and nonjudgmental for sharing experiences and getting peer support [34]. Social media can be a living laboratory for studying algorithms for harmful trends, such as acute suicide ideation [35]. Studies of why people use social media and the nature of social media addiction inevitably point toward the psychological and behavioral characteristics of the users [36,37].

If we are to learn from Molly’s tragedy, we must understand what she was doing online and why she was doing it. From the standpoint of a social media user, aren’t most social media sessions some form of self-therapy? Aren’t adolescents looking for help, reassurance, and support before they are sucked into the horrifically dark content on these platforms? How different are sessions on social media from sessions on DTx? The interfaces have a lot of similarities. The mechanisms and rewards are similar. For adolescents, is social media use a form of digital therapeutic? When a child is alone, upset, bored, angry, or unable to sleep, is it not too easy to reach for a screen and dive into the metaverse? Are their sessions not filled with content, graphics, games, and surveys? Are they not seeking the same endorphin hit reward that keeps patients engaged in DTx therapies?

We know social media is addictive and can isolate users from their families and physical environments [38]. We know it can radicalize susceptible people toward mass violence [39]. Yet we treat these platforms like ATM machines for advertisers, like they are grandma’s family picture albums. They are a free and open data set for every form of unconsented social, economic, and psychological analysis and tracking conceivable. Yet they remain entirely unregulated from the standpoint of the types of mental and physical harm they cause.

## Current Social Media Regulation

The core challenge is that addiction and behavioral influence *are* the social media business models [40]. Addiction is a severe health concern to parents and family members, but it is a feature of these platforms. The core business model of social media is the generation of revenue via the sale of advertising [14,40]. Just as television and radio industries grew by providing content funded by advertisers, social media has evolved similarly. The critical difference is that social media is not a one-way content delivery but a deeply immersive interaction and experience. Early television viewers did not provide “free” services encouraging users to store large amounts of their personal information. Further, early television and radio could not track the use patterns of individual users, let alone log every touch of a button, as technologies such as keyloggers require 2-way communication [41]. Given the significant differences and similarities, it makes sense for the federal agencies that regulate television, such as the Federal Communication Commission in the United States, to regulate social media via legislations such as the Communications Act [42]. Similarly, the Federal Trade Commission in the United States has some jurisdiction over social media because of its authority over privacy. It has also levied the greatest fine ever against a social media company via a US $5 billion penalty and new restrictions [43].

In the United Kingdom, many call for an acceleration of the approval of the Online Safety Bill in response to the Molly Russell tragedy. Still, some privacy experts are concerned that the bill attacks free speech and uses essential security capabilities such as encryption [44]. This has been a challenge with current regulatory regimes and social media. From privacy to disinformation, the protections sought by some are instantly perceived as an invasion of privacy or an assault on the rights of others.

Are online privacy laws and the Communications Act the best vehicles for social media regulation? Even with the current regulations, how can these platforms commit offenses that can harm consumers? Given Molly’s case and the growing body of evidence on the underlying health harms, pathologies, and etiologies, we propose that medical benefit-risk assessment be given the highest priority.

## Regulating Social Media as a Digital Therapeutic

The regulation of DTx is quite complex. There can be very unclear lines between prescription therapeutics having regulatory approval and the many wellness apps available to consumers of all ages. Since the introduction of mobile phones and social media, suicide rates in children and adolescents have been on the rise and have increased during the COVID-19 pandemic, driving a significant unmet need for solutions of all types [37]. One of the opportunities and challenges of current DTx is that they are relatively low-risk interventions, but most require a prescription [45]. Given the huge unmet need, some feel that DTx would have the potential to bridge gaps in the continuum of care if psychologists were permitted to prescribe them. Indeed, if social media is one large DTx environment, for good or bad, how can it be harnessed for good? Some solutions are as follows:

- Accountable content moderation is needed, with steep fines and loss of revenue for harmful content. We know these platforms are out of control. Reign them in or shut them down. Most of this content is far less subtle than political misinformation. Images of suicide, violence, and self-mutilation should be removed, *and* the threat actors must be identified and prosecuted when possible.
- Equal time should be given for beneficial content via ad targeting effectively within known networks. Have filters that drive content to contacts and family members. They are already doing this despite their professed privacy policies. We have public service ads on television; we can extend that model to require social media companies to do more to promote warnings and sources of help from vetted and trusted sources. Do it in service of good, not just profits.
- Partner with governments and NGOs to combat anxiety, depression, and suicide. Linking individuals with services such as crisis text lines and other crisis service providers in real-time to provide the possibility of help 24/7 and 365 days a year are required. We would need to have a process to review the providers and ensure they have the resources to respond to calls. We have spent billions bailing out the banks. Why can we not spend billions saving our children?
- Social media companies should fund and develop an international registry of adverse events, just as a pharmaceutical company must build, maintain, and share detailed adverse event and safety data on all their authorized products. Incidents of harmful content and the implications of that should be reportable by users, health providers, and parents, and the government must investigate them. We do not allow the private industry to self-regulate on harmful adverse events to medicinal products. Why should we let social media companies self-regulate?
- Comprehensive regulation is required. Due to a lack of jurisdiction and inadequate historical precedent, the fragmented regulatory landscape has enabled multiple harms to persist. In the United States, the Federal Communication Commission, Federal Trade Commission, FDA, Department of Justice, and Department of Homeland Security should build and enact a comprehensive regulatory approach. In the European Union, the lines are somewhat clearer, but a similar approach would help. Indeed, even Mark Zuckerberg has gone on record that new regulatory paradigms are needed [46]. The fragmentation needs to end, and we need a lead agency to lead and coordinate the efforts of the other agencies. This is why we created organizations like Federal Emergency Management Agency to lead in a catastrophe. We have a catastrophe with mental health and social media, and no one is leading.
- More basic, translational, and applied research should be carried out. There is so much to learn in this area that a genuinely focused research strategy is needed and perhaps should be a priority for the new Advanced Research Projects Agency for Health. We have used artificial intelligence and extensive data collection to detect a potential terrorist attack on our nation. It is time to fund artificial intelligence–tailoring algorithms to detect harmful websites that encourage youth to harm themselves and react the same way as those other terrorist threats. The threat is as large and as real as it gets.
- Targeted but prolific public awareness campaigns are needed. Despite recent failures in public health messaging that plagued the COVID-19 pandemic, there have been highly effective national public health awareness campaigns [47]. The role of public health agencies and universities needs to be revisited. During the pandemic, few public health agencies had coordinated and effective communication strategies to help guide public behavior. Universities do excellent research, but their role in actively engaging in public health communication needs to increase beyond publishing excellent academic papers. This may require more funding and academic credit for public service functions. Qualified people should fill these positions, not political or personal patronage appointments.

Implementing these changes will require collaboration across government agencies and public inputs. Although this may be complex and take time, it needs to be done with urgency, given the broad impact this has on our youth and its long-term consequences. Both the public and private sectors can play a role in making sure this becomes a national priority. There are some previous precedents; for example, public policies have been developed for advertising for children, but this will require more complex strategies given the global scope of internet sources.

**Figure 2:**
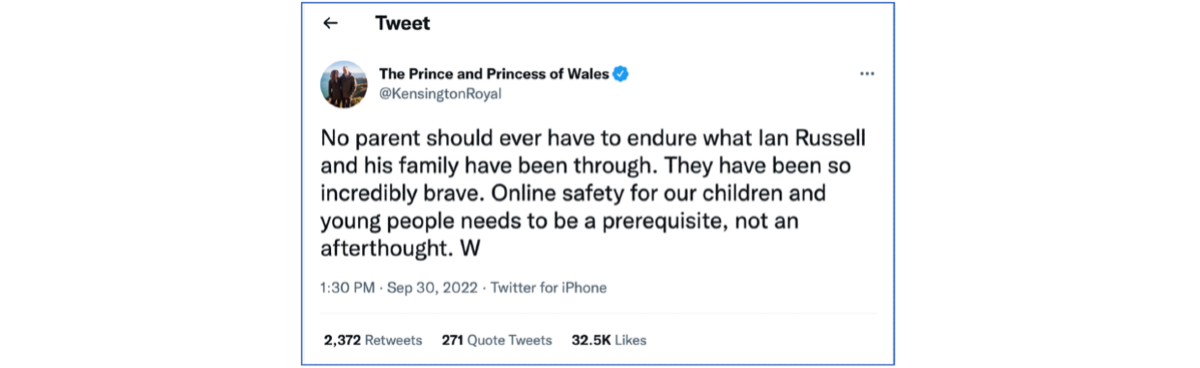
The Prince and Princess of Wales' tweet on September 30, 2022 [48].

Lastly, the best way to change society is to be part of the change we want. We cannot wait for others to act. We all must act. We need to make our voices heard. We need to question how the organizations we lead are contributing to the problem, and if we do not have the resources, we need to ask for those resources loudly. We need to expect more from our leaders in government and businesses to do more and have visible results. We need to hold people accountable when they have posted harmful content or failed to act. We need to own the problem and do something about it, not just hope that someone else will fix it. We do not need photo ops of leaders expressing sympathy. We need a plan with tangible, measurable outcomes, target dates, a responsible agency, and leaders to implement an action plan. Perhaps the Prince and Princess of Wales have already said it better than we did (Figure 2) [48,49].

## Abbreviations

CBT: cognitive behavioral therapy
DTx: digital therapeutics
FDA: Food and Drug Administration
